# Naturally circulating pertactin-deficient *Bordetella pertussis* strains induce distinct gene expression and inflammatory signatures in human dendritic cells

**DOI:** 10.1080/22221751.2021.1943537

**Published:** 2021-07-05

**Authors:** Michiel M. Kroes, Alberto Miranda-Bedate, Elise S. Hovingh, Ronald Jacobi, Corrie Schot, Elder Pupo, René H. M. Raeven, Arno A. J. van der Ark, Jos P. M. van Putten, Jelle de Wit, Rob Mariman, Elena Pinelli

**Affiliations:** a Center for Infectious Disease Control, National Institute for Public Health and the Environment, Bilthoven, Netherlands; b Department of Infectious Diseases and Immunology, Faculty of Veterinary Medicine, Utrecht University, Utrecht, Netherlands; c Institute for Translational Vaccinology (Intravacc), Bilthoven, Netherlands

**Keywords:** Pathogen adaptation, pertactin, dendritic cell, transcriptomics, RNAseq, proteomics, innate immunity, human

## Abstract

Respiratory infections caused by *Bordetella pertussis* are reemerging despite high pertussis vaccination coverage. Since the introduction of the acellular pertussis vaccine in the late twentieth century, circulating *B. pertussis* strains increasingly lack expression of the vaccine component pertactin (Prn). In some countries, up to 90% of the circulating *B. pertussis* strains are deficient in Prn. To better understand the resurgence of pertussis, we investigated the response of human monocyte-derived dendritic cells (moDCs) to naturally circulating Prn-expressing (Prn-Pos) and Prn-deficient (Prn-Neg) *B. pertussis* strains from 2016 in the Netherlands. Transcriptome analysis of moDC showed enriched IFNα response-associated gene expression after exposure to Prn-Pos *B. pertussis* strains, whereas the Prn-Neg strains induced enriched expression of interleukin- and TNF-signaling genes, as well as other genes involved in immune activation. Multiplex immune assays confirmed enhanced proinflammatory cytokine secretion by Prn-Neg stimulated moDC. Comparison of the proteomes from the Prn-Pos and Prn-Neg strains revealed, next to the difference in Prn, differential expression of a number of other proteins including several proteins involved in metabolic processes. Our findings indicate that Prn-deficient *B. pertussis* strains induce a distinct and stronger immune activation of moDCs than the Prn-Pos strains. These findings highlight the role of pathogen adaptation in the resurgence of pertussis as well as the effects that vaccine pressure can have on a bacterial population.

## Introduction

The respiratory disease pertussis, also known as whooping cough, is a human specific infection caused by the Gram-negative bacterium *Bordetella pertussis*. This highly contagious disease is characterized by severe and persistent cough in all age groups but can be fatal in unvaccinated or not fully vaccinated infants [1,2]. A whole cell pertussis vaccine was introduced worldwide in the 1950s, which resulted in a drastic drop in pertussis notifications. Due to adverse effects, the whole cell pertussis vaccine was replaced in most industrialized countries by the safer acellular pertussis (aP) vaccine since the 1990s. Despite high vaccination coverage in countries using aP vaccination, pertussis cases have been increasing in the last three decades [3]. Several reasons for this increase have been suggested, including waning of vaccine-induced immunity and suboptimal induction of a protective type of immune response by the aP vaccine [4,5]. In addition, genetic changes of *B. pertussis* due to external factors such as vaccination, also referred to as bacterial adaptation, may play a role in the reemergence of this disease [6]. One of the most pronounced genetic changes observed in *B. pertussis* after the introduction of the aP vaccine is the loss of pertactin (Prn) expression, which is one of the aP vaccine components. Prn is an autotransporter protein of *B. pertussis* that is auto-cleaved from the outer membrane, and has been identified as a minor adhesin [7,8] with immunomodulatory properties [9,10]. The aP vaccine induces a strong antibody response directed against Prn and these antibodies play a critical role in opsonophagocytosis and complement mediated killing of *B. pertussis* [11,12]. These anti-Prn bactericidal antibodies have been suggested to drive the emergence of Prn-deficient *B. pertussis* strains [6]. The percentage of circulating *B. pertussis* strains that are deficient in Prn is rapidly increasing worldwide reaching up to 90% of the circulating *B. pertussis* strains in some countries [13–15]. It has recently been documented that the longer the period since the introduction of a Prn-containing aP vaccine, the higher the prevalence of circulating *B. pertussis* strains that do not express Prn [13].

Dendritic cells (DCs) are innate immune cells that sense the presence of invading microorganisms via pattern recognition receptors (PRRs) which recognize evolutionary conserved structures of pathogens [16]. Activation of PRRs on the DCs results, among others, in the secretion of diverse cytokines. Cytokine secretion and antigen presentation by DCs play a crucial role in orchestrating the adaptive immune response, making these cells essential in bridging the innate and the adaptive immune system [17].

In a previous study using Prn-knockout (KO) *B. pertussis* and their isogenic strains, we showed that in the absence of this virulence factor *B. pertussis* induces enhanced proinflammatory cytokine secretion by moDC [9]. The aim of the current work was to determine whether naturally circulating Prn-deficient *B. pertussis* strains also induce a different DC response as compared to their Prn-expressing counterparts. We also investigated whether Prn is the only differentially expressed protein in the Prn-deficient *B. pertussis* strains. To this end, a selection of genetically matched Prn-expressing (Prn-Pos) and Prn-deficient (Prn-Neg) *B. pertussis* strains circulating in the Netherlands in 2016 were used to stimulate monocyte-derived DCs (moDCs). Additionally, Prn-Pos strains isolated in 1998 were included to investigate whether the year of *B. pertussis* isolation has an effect on moDC activation. The *B. pertussis* strains used were further characterized by determining their proteomic profile using whole cell liquid chromatography-mass spectrometry (LC-MS). The activation of human moDCs by these live *B. pertussis* strains was investigated by analyzing their cytokine secretion and determining their transcriptome using RNA sequencing (RNAseq). The applied strategy of combining multiple omics-techniques that is gaining popularity [18,19] provided unbiased information on both host response (RNAseq and cytokine profiling) and bacterial pathogen composition (LC-MS). Findings indicated distinct moDC activation by Prn-Neg *B. pertussis* strains when compared to Prn-Pos strains. Moreover, we found specific bacterial protein expression profiles associated with the Prn status of the *B. pertussis* strains.

## Material and methods

### Ethics statement

This study was conducted according to the principles described in the Declaration of Helsinki. Buffy coats were provided by the Sanquin Blood Supply. For the collection of samples and subsequent analyses, all blood donors provided written informed consent. Blood samples were processed anonymously and the research goal, primary cell isolation, required no review by an accredited Medical Research Ethics Committee, as determined by the Dutch Central Committee on Research involving human subjects.

### Bacterial strains and growth conditions

For more details about strain selection see supplementary Materials & Methods (Table S1). To ensure the consistent use throughout the different experiments of *B. pertussis* strains that are at the same growth phase, flash freeze vials were prepared as previously described [20].

### HEK-Blue TLR assay

In order to quantify human (h)TLR2 and hTLR4 activation by the various *B. pertussis* strains the NF-κB/SEAP reporter HEK293-Blue (HEK-Blue) cell lines expressing hTLR2 (HEK-Blue-hTLR2) or hTLR4 (HEK-Blue-hTLR4) were used (InvivoGen) as previously described [21].

### Determination of *B. pertussis* virulence factors using whole-cell ELISA

Whole-cell ELISA was used to measure the production of various virulence factors, namely Prn, Ptx, FHA and Vag8, by the selected *B. pertussis* strains. Whole-cell ELISA was performed as previously described [9] with minor alterations as described in supplementary Materials & Methods.

### Purification, culture and stimulation of moDCs

Buffy coats from healthy human donors were used for the isolation of CD14 positive monocytes for the subsequent generation of moDCs as previously described [21]. The purity of the CD14 positive cells was at least 95%. On day 6, the immature moDCs were exposed to the indicated *B. pertussis* strains, taken from the flash freeze vials (MOI 10), in DC culture medium supplemented with 500 U/ml GM-CSF or left without bacteria for 3 h for RNA isolation or for 24 h for cytokine determination.

### Cytokine analysis by luminex

The concentration of various cytokines (IL-6, IL-8, IL-10, IL-12p70, G-CSF, TNF) in supernatants of moDC was determined using Bio-plex Pro cytokine kits (Bio-Rad) according to the manufacturer’s instructions. All supernatants were filtered over a 0.22 μm filter to remove bacteria before cytokine determination (Millipore).

### RNASeq analysis and data processing

Total RNA was extracted from the moDCs cultured in the presence of the indicated stimuli after 3 h (for more details, see supplementary Materials & Methods). RNAseq reads were aligned to the Homo sapiens reference genome (GRCh38) and most recent transcript annotations using STAR (2.6.1a). Raw counts were filtered for low count genes, eliminating those targets with less than 1 count in at least 10 samples, and full quantile normalized (*EDAseq*) *RUVr* from *RUVseq* R package was employed to obtain the covariates that correct for Donor and Batch effects in the downstream analysis.

### Differential gene expression analysis and pathway enrichment analysis

The differentially expressed gene (DEG) lists were obtain with *DeSeq2* R package under standard parameters, for the comparisons Unstim-Ctrl vs Prn-Pos (Table S2), Unstim-Ctrl vs Prn-Neg (Table S3) and Prn-Pos vs Prn-Neg (Table S4). Genes were considered significantly expressed if they showed an adjusted *p*-value (*Bonferroni-Hochberg* multiple testing (padj)) lower than 0.05. Heatmaps to represent these DEGs were generated with the aid of *ComplexHeatmap* R package. Dendrogram trees were obtained by hierarchical clustering (*Ward’s D2* method) of *spearman* correlation gene distances or K-means supervised clustering. K was selected based on the number of groups involved in the analysis (Prn-Neg, Prn-Pos and/or Unstim-Ctr).

Pathway enrichment analysis was performed with *fgsea* R package. DEG and no DEGs were given as input, ranked by the following formula: −log10(padj) * signed log2(Fold Change). *ggplot2* R package (Figure 3(b)) and *Cytoscape* software version 3.8.2 [22] (Figure 3(c,d)) were used for the representation of the results.

For the DC focused analysis in Figure 4 we took advantage of the Blood Transcription Modules (BTM), as defined by Li et al. 2014 [23]. A total of 234 genes (Table S5) were found to be associated with 13 different DC processes, selected by the presence of keywords “dendritic” and/or “DC” in their composite name. In addition to the DC associated BTM, genes encoding for the measured cytokines in supernatant as well as Toll-like receptors (TLR) and NOD receptors were included when missing, for the completeness of the analysis.

### Protein–Protein interaction network analysis

All DEGs from the GSEA analysis were used as input for *STRING* (confidence > 0.7, https://string-db.org). The resulting interactome was processed and visualized with *Cytoscape* software version 3.8.2 [22], annotated with *AutoAnnotate* app (v.1.3.3, http://www.baderlab.org/Software/AutoAnnotate). MCL clustering under standard parameters was used, and singletons and couples were eliminated for Figure 3(c,d).

### Liquid chromatography–mass spectrometry (LC–MS) and data processing

LC–MS/MS proteomics analysis was performed as previously described [24], except that peptides were not chemically labelled. For peptide identification and corresponding proteins, MS/MS spectra were searched against the protein database of *B. pertussis* Tohama (NCBI 257313) (3258 entries) as previously applied [25].

The differential expressed proteins (DEP) list (Table S6) was obtained by the robust summarization method from the *msqrobsum* R package [26], following author’s pipeline (https://github.com/statOmics/MSqRobSum/blame/master/vignettes/msqrobsum.Rmd). *ggplot2* R package was employed for the heatmaps, and *plot()* function from *base* R package for the volcano plot (Figure 5). The input for these graphs were the robust summarized protein signals, considering them as DEP if the Bonferroni-Hochberg padj was lower than 0.05 and the fold change was higher than 1.5.

### Statistical analysis

Linear models or permutation tests were employed for the statistical analysis, after assumptions were assessed, and effect sizes (ES) were calculated, *Hedges’g* [27] and Cliff’s delta [28], where applied (for more details, see supplementary Materials & Methods). All comparisons with a padj < 0.05 and ES medium or higher are considered as biologically relevant in the present work.

For all analysis and plots, unless specifically indicated, R software (v4.03) was used.

## Results

### Production of virulence factors and TLR2/4 activation by the selected *B. pertussis* strains

**Figure 1.**
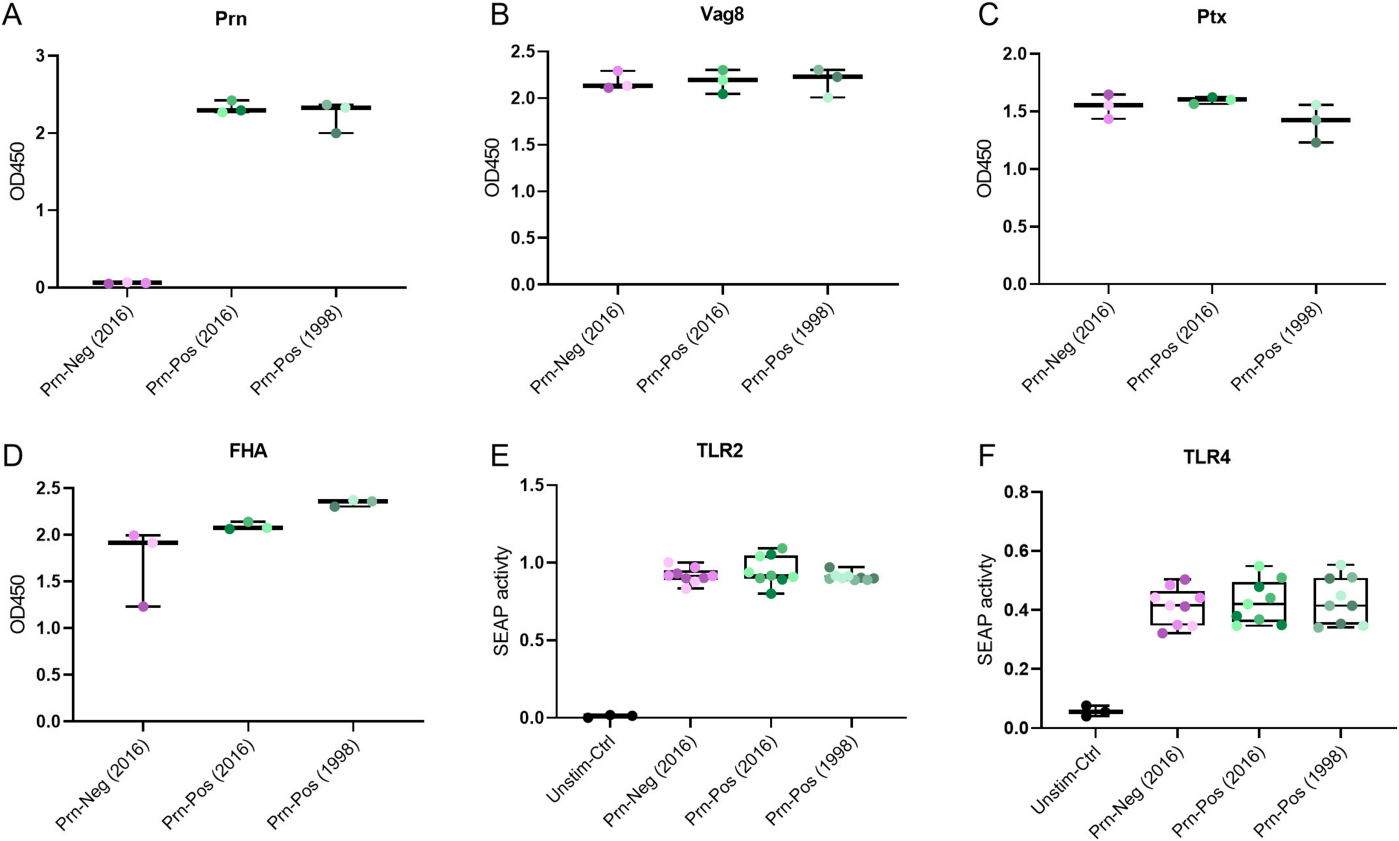
*B. pertussis* strain characterization. Protein levels of (a) Prn, (b) Vag8, (c) Ptx and (d) FHA were determined for all nine *B. pertussis* strains using whole-cell ELISA. (e) TLR2 and (f) TLR4 activation in HEK blue cell lines with the nine live *B. pertussis* strains (MOI 40) or their respective ligands, PAM3CSK4 (100 ng/ml) for TLR2 and LPS-EK (10 ng/ml) for TLR4. The represented SEAP activity is normalized against the SEAP activity induced by the respective TLR ligands. Dots indicate individual measurements. Colours in dots indicate different *B. pertussis* strains (Table S1): A = dark purple, B = purple, D = light purple, F = dark green, G = green, H = light green, J = dark grey-green, K = grey-green, L = light grey-green. Data is representative of at least 3 independent experiments.

To investigate the effect that Prn deficiency in *B. pertussis* has on moDC activation, nine *B. pertussis* strains (Table S1) isolated from Dutch pertussis patients were selected. We characterized the production of several virulence factors and TLR2/TLR4 activation by these *B. pertussis* strains. As expected, six of the *B. pertussis* strains produced Prn (Prn-Pos) whereas the three other strains lacked Prn production (Prn-Neg) (Figure 1(a)). All nine strains expressed comparable levels of Virulence Associated Gene 8 (Vag8) (Figure 1(b)) as well as other antigens present in the aP, namely Ptx (Figure 1(c)) and Filamentous Hemagglutinin Adhesin (FHA) (Figure 1(d)). These nine *B. pertussis* strains also showed comparable TLR2 (Figure 1(e)) and TLR4 (Figure 1(f)) activation. These data indicate that the major difference among the selected strains observed so far, is the expression of Prn.

### Prn-Neg *B. pertussis* induces enhanced moDC cytokine secretion

**Figure 2.**
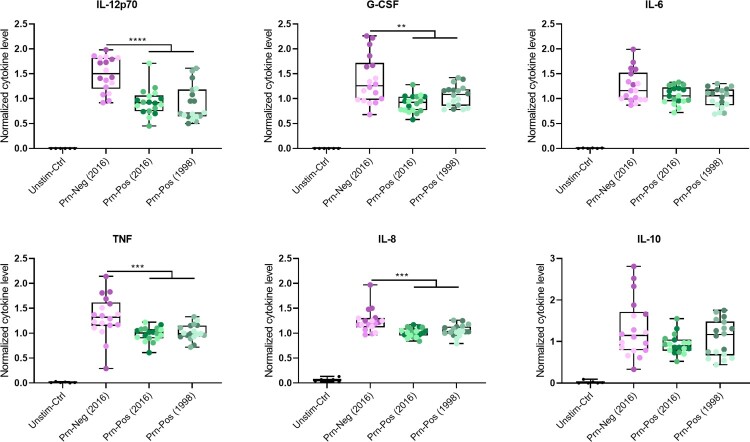
Increased cytokine secretion of moDCs stimulated with Prn-Neg *B. pertussis* strains compared to the stimulation with Prn-Pos strains. moDCs cytokine secretion was measured after stimulation with strains A, B, D (Prn-Neg (2016)), F, G, H (Prn-Pos (2016)), J, K or L (Prn-Pos (1998)), or untreated controls (Unstim-Ctrl) for 24 h. Cytokine values in y axes were normalized against the mean of all measured concentrations of that cytokine. Dots indicate individual donors. Colours in dots indicate different *B. pertussis* strains (Table S1): A = dark purple, B = purple, D = light purple, F = dark green, G = green, H = light green, J = dark grey-green, K = grey-green, L = light grey-green. Results are represented as boxplots showing normalized cytokine levels. **p <* *0.01, ***p <* *0.001, ****p <* *0.0001. Significance is represented when the effect size is ≥Medium (Table S7).

To assess whether Prn-deficient *B. pertussis* strains had a distinct effect on the cytokine secretion by moDCs compared to their Prn-expressing counterparts, we stimulated moDC from 6 donors with the nine live *B. pertussis* strains for 24 h. All *B. pertussis* strains induced secretion of IL-12p70, TNF, G-CSF, IL-8, IL-6 and IL-10 compared to unstimulated (Unstim-ctrl) moDCs (Figure 2). Analysis of the normalized data shows significantly (Table S7) higher level of cytokine secretion by moDCs stimulated with Prn-Neg *B. pertussis* strains compared to Prn-Pos strains for IL-12p70, TNF, G-CSG and IL-8 (Figure 2, Table S8). No significant difference in cytokine secretion was observed between moDCs stimulated with the Prn-Pos *B. pertussis* strains isolated in 1998 vs. 2016 (Figure 2). These findings suggested an enhanced proinflammatory cytokine secretion by moDC upon stimulation with the Prn-Neg *B. pertussis* strains compared to the Prn-Pos strains.

### Prn-Neg *B. pertussis* induces distinct gene expression profiles in human moDCs

**Figure 3.**
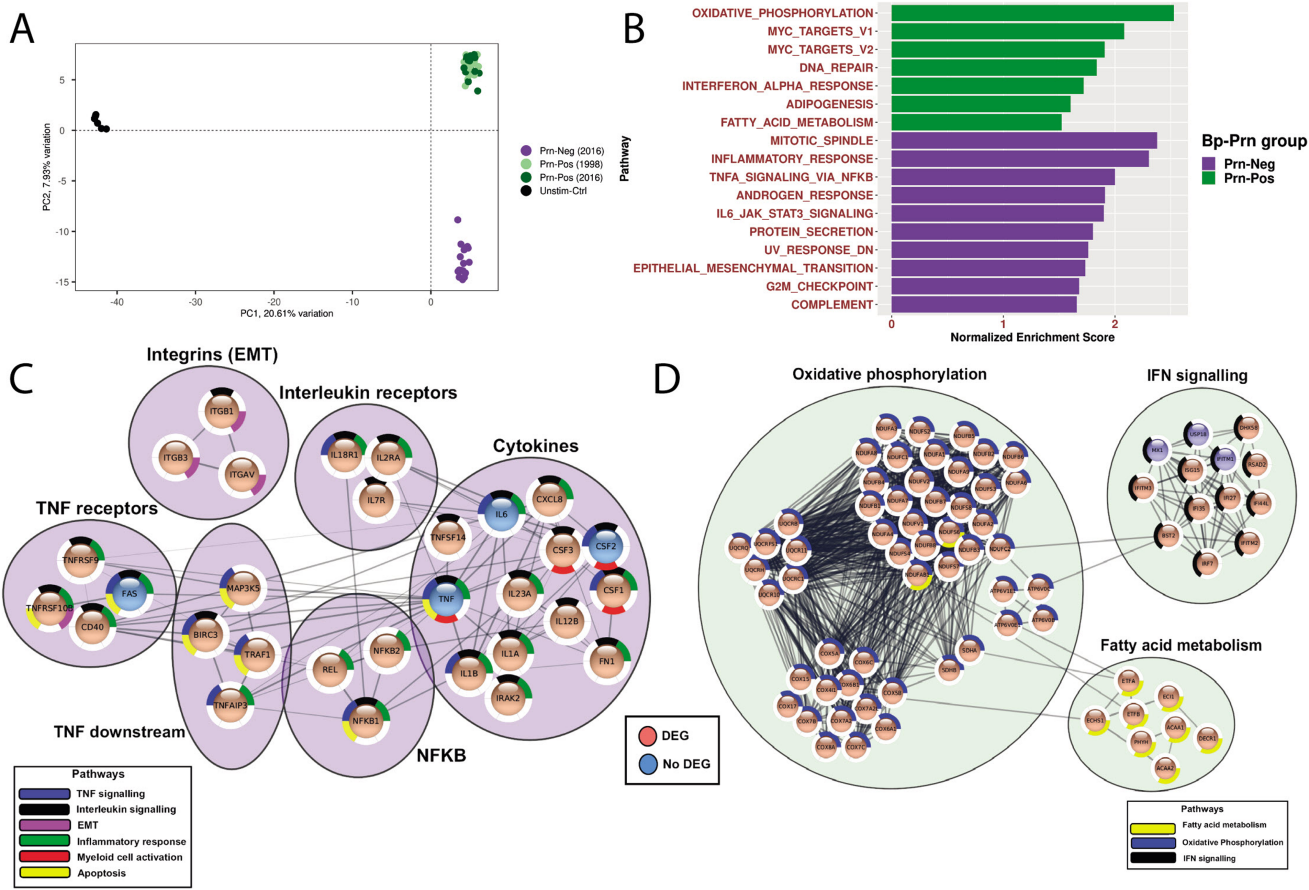
Distinct gene expression profile in Prn-Neg stimulated moDCs. (a) moDCs were unstimulated (black) or stimulated with 3 different Prn-Neg *B. pertussis* strains (purple) and 6 Prn-Pos *B. pertussis* strains (dark green, isolated in 2016; light green, isolated in 1998). Normalized corrected read counts from all RNAseq genes were analyzed with PCA. (b) Gene set enrichment analysis (GSEA) of *B. pertussis*-stimulated moDCs. Green bars represent pathways enriched in Prn-Pos, purple bars represent enrichment in Prn-Neg stimulated moDCs. (c, d) Protein-protein interaction clusters of all genes included in GSEA pathways from (b), of moDCs after (c) Prn-Neg (purple) or (d) Prn-Pos (green) stimulation; DEG, differentially expressed genes (padj < 0.05, Fold Change > ±1.2) between Prn-Pos and Prn-Neg stimulated moDCs; EMT, epithelial to mensenchymal transition.

Since immunomodulatory properties were previously described for Prn [9,10], we hypothesized that the DC response to *B. pertussis* could be altered in its absence. For this purpose, also the gene transcription profile of moDCs after 3 h of *B. pertussis* stimulation or that of unstimulated cells were obtained by RNAseq. After correcting for donor and batch effects, principal component analysis (PCA) of the normalized read counts separately clustered the Unstim-Ctrl and *B. pertussis*-stimulated moDCs (PC1 21.61%). Moreover, PC2 revealed that the *B. pertussis*-stimulated moDC cluster was composed of two smaller clusters based on the Prn expression of the *B. pertussis* strains (PC2 7.93%), indicating subtle but clear differences between the Prn-Pos and Prn-Neg group (Figure 3(a)). Since we again did not observe any difference between moDCs stimulated with Prn-Pos strains from 1998 or 2016 we grouped all Prn-Pos strains for further analysis.

To determine gene expression differences in moDCs upon Prn-Pos or Prn-Neg *B. pertussis* stimulation, we compared normalized gene counts in the Prn-Pos vs the Prn-Neg group. This analysis revealed 134 genes that were significantly higher expressed in the Prn-Pos group and 289 genes in the Prn-Neg group (padj < 0.05; Fold change > 1.2, Table S4). Prn-Neg stimulated moDCs showed significantly higher expression of *CSF3, CXCL8* and *IL12B*, encoding for G-CSF, IL-8 and IL-12p40 respectively (Table S4). This is in accordance with the enhanced secretion of these cytokines observed in Figure 2, validating our RNAseq data as a valuable proxy for protein translation.

To better understand which biological processes are differentially induced in moDCs by Prn-Pos and Prn-Neg *B. pertussis* strains, we performed a gene set enrichment analysis (GSEA) [29] to identify gene enrichment in various pathways. For the Prn-Neg stimulated moDCs, this analysis revealed an enriched gene expression in pathways associated with interleukins (JAK-STAT pathway), TNF-signaling via NF-κB, proliferation (G2/M checkpoint; Mitotic spindle) and inflammatory responses (Figure 3(b,c)). This is consistent with the enhanced proinflammatory cytokine secretion observed by moDCs stimulated with Prn-Neg *B. pertussis* strains (Figure 2). In contrast, the Prn-Pos *B. pertussis*-stimulated moDCs exhibited an enriched gene expression associated with an IFNα immune response, oxidative phosphorylation and fatty acid metabolism (Figure 3(b,d)). Looking into concrete differentially expressed genes associated with these pathways, not only the expected *IL12B*, *CSF3* and *CXCL8* were higher expressed in the moDC stimulated with the Prn-Neg strains, but also other proinflammatory cytokines such as *IL23A*, *IL1A, IL1B* and *TNFSF15* (Figure 3(c), Table S4). These data further corroborate the observation that Prn-Neg *B. pertussis* induces an overall stronger proinflammatory immune response by moDCs. This response is characterized by a proliferative and an interleukin/TNF skewed transcriptional phenotype, which is different from the IFNα skewed transcriptional phenotype induced by Prn-Pos *B. pertussis*.

As the observed gene expression appeared to be a good proxy for protein expression, all DEGs from the GSEA pathways were submitted to STRING for a protein–protein interaction analysis. We retrieved several dense interacting clusters for Prn-Neg stimulated moDCs, such as cytokines, TNF related (receptors, NFκB and downstream targets) and integrins that are representative of epithelial to mesenchymal transition (Figure 3(c)). TNF is highly connected with the various clusters, indicating a prominent role for TNF in the enriched pathways for the Prn-Neg stimulated moDCs. In contrast, for Prn-Pos stimulated moDCs oxidative phosphorylation, IFN signalling and fatty acid metabolism related clusters (Figure 3(d)) were formed. In both cases the clusters represent the major functional groups of GSEA analysis described above. This suggests that the genes belonging to the pathways described in Figure 3(b) were not only transcriptionally enriched, but that they also interact at the protein level.

### Distinct DC associated blood transcription module gene expression

**Figure 4.**
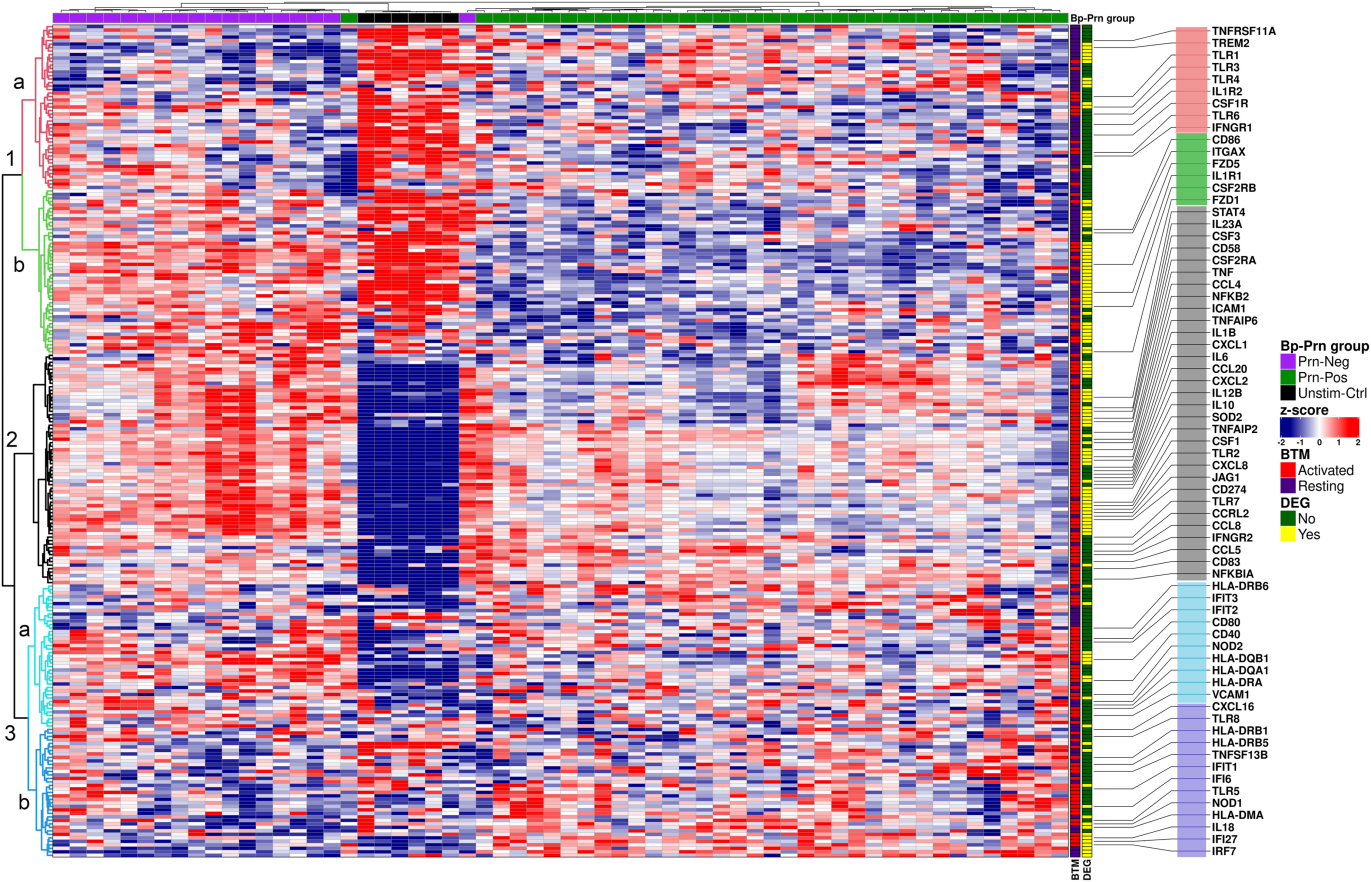
DC associated Blood Transcription Modules (BTM) confirmed an over-representation of immune activated genes for Prn-Neg stimulated moDCs compared to the stimulation with Prn-Pos strains. Heatmap representing a hierarchical clustering of all DC associated BTM gene counts (rows; *z*-scores, scaled and centered per gene) per individual moDC donors, stimulated with different *B. pertussis* strains (columns, Unstim-Ctrl moDCs (black); Prn-Pos (green) and Prn-Neg (purple) stimulated moDCs). *BTM* labeling represents genes associated with “Resting” (blue) or “Activated” (red) DC-related BTMs; *DEG* labeling, differentially expressed genes (padj < 0.05, Fold Change > ±1.2) between Prn-Pos and Prn-Neg stimulated moDCs. An educated selection of important immune related genes is shown in rows for better visualization. For a complete list, see Table S5.

Next, we analyzed the moDC gene expression as defined by blood transcription modules (BTM) [23]. BTM are groups of genes (modules) associated with a specific immune function and/or cell type. A supervised hierarchical clustering of the DC associated BTM showed, a clear separation between Unstim-Ctrl, Prn-Pos and Prn-Neg stimulated moDCs (Figure 4). Moreover, three major clusters of genes were revealed. Cluster 1 showed predominantly genes annotated as “genes expressed by resting DCs” in the BTM. Cluster 1a (Figure 4, pink in the dendrogram) is composed of genes such as several TLRs and cytokine receptors (*IL1R2*, *CSF1R* and *IFNGR1*) that were predominantly expressed in Unstim-Ctrl moDCs. Cluster 1b (Figure 4, green in the dendrogram) mainly contained genes highly expressed in Unstim-Ctrl and Prn-Neg stimulated moDCs, and included genes encoding immune receptors such as *IL1R1* and *CSF2RB*, the costimulatory molecule *CD86* and the integrin alpha X (*ITGAX* or CD11c). Cluster 2 (Figure 4, black in the dendrogram) almost exclusively contained genes associated with activated DCs. These genes were highly expressed after Prn-Neg stimulation while they showed only intermediate expression in Prn-Pos stimulated moDCs. Unstim-ctrl moDCs showed little to no expression of these cluster 2 genes. This cluster not only included many genes encoding for secreted cytokines (*IL23A*, *CSF3*, *TNF*, *IL1B*, *IL6*, *IL12B*, *IL10* and *CSF1*) and chemokines (*CCL4*, *CXCL1*, *CCL20*, *CXCL2*, *CXCL8*, *CCL8* and *CCL5*) but also genes involved in NFκB signaling (*NFKB2* and *NFKBIA*). This is in accordance with the previous GSEA, which highlighted the enrichment of interleukin/TNF signaling, protein secretion and inflammatory responses in the Prn-Neg stimulated moDCs (Figure 3(b)). Cluster 3 again predominantly contained genes associated with activated DCs. Cluster 3a (Figure 4, light blue in the dendrogram) was mainly expressed in Prn-Neg stimulated moDCs and contained genes associated with antigen presentation (*HLA-DQ(A1 and B1)*, *HLA-DR(B6 and A)*) and co-stimulatory molecules (*CD40* and *CD80*). Genes in cluster 3b (Figure 4, magenta in the dendrogram) showed the highest expression in Prn-Pos stimulated moDCs. This cluster included many genes induced by IFNα (*IFI6*, *IFIT1*, and *IFI27*) or involved in the regulation of IFNα associated processes (*IRF7*). These data again indicate that Prn-Pos *B. pertussis* induced a dominant IFNα type of immune response in moDCs. Overall, Prn-Neg stimulated moDCs showed enhanced gene expression of proinflammatory cytokines, chemokines, and antigen presentation molecules, indicating a stronger proinflammatory activation than the Prn-Pos stimulated moDCs.

### Distinct protein expression profile of Prn-Neg *B. pertussis* strains

**Figure 5.**
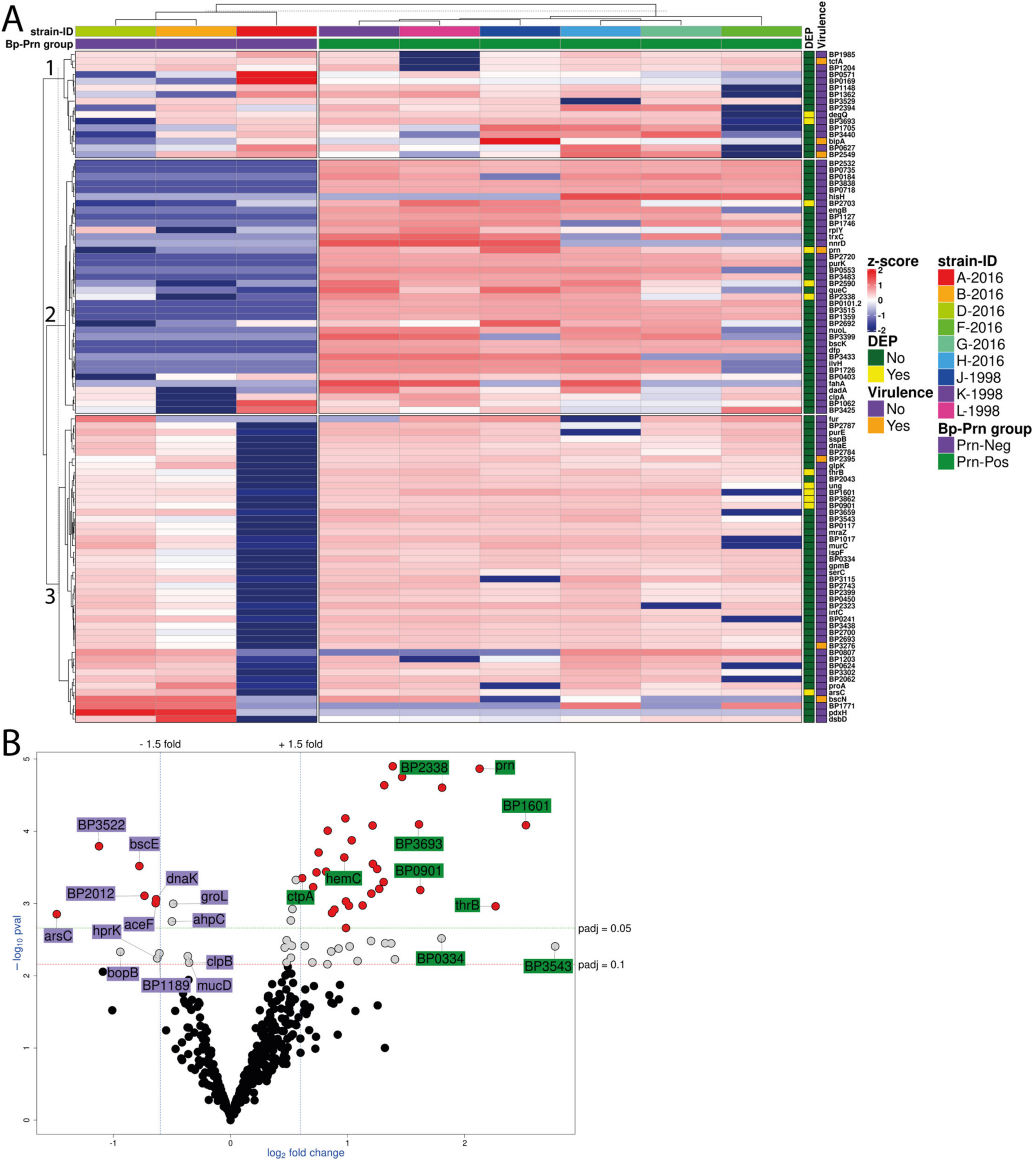
Protein expression analysis with LC-MS showed a differential proteomic profile between *B. pertussis* Prn-Neg and Prn-Pos strains apart from pertactin deficiency. (a) Heatmap representing a hierarchical clustering of the protein expression (*z*-scores, scaled and centered per protein) from the top 100 most variable proteins (rows) per individual *B. pertussis* strains (columns). *DEP* labeling indicates significant differentially expressed proteins (padj < 0.05, Fold change > ±1.5). *Virulence* labeling indicates whether the protein is defined as Bvg-regulated according to Streefland et al. [42]. (b) Volcano plot of DEPs upregulated in Prn-Pos (green squared proteins) and in Prn-Neg (purple squared proteins) *B. pertussis* strains. Red dots represent DEPs (padj < 0.05, Fold change > ±1.5); grey dots represent proteins above the padj = 0.1 cut off but below the padj = 0.05 and/or 1.5 fold change cut off; black dots represent proteins below the padj = 0.1 cut off.

It has previously been suggested that the loss of Prn in *B. pertussis* is accompanied by other compensating mutations which are essential for the fitness of Prn-deficient *B. pertussis* strains [9,14,15] and consequently, can also affect the activation of immune cells. In order to determine whether specific changes in protein expression are associated with Prn-Neg strains, we performed proteomics analysis of whole cell lysates of all nine *B. pertussis* strains by LC-MS. K-means supervised hierarchical clustering of the top 100 most variable expressed proteins clustered the Prn-Neg strains apart from the Prn-Pos *B. pertussis* strains (Figure 5(a)). This indicates that, besides Prn expression, there are other proteins whose expression varied between the two Prn groups. Especially, cluster 2 contains many proteins which show high expression in the Prn-Pos strains but very low expression in the Prn-Neg strains. No clear function could be ascribed to this group of genes. Only a few virulence factors were found to be differentially expressed, namely bscE, involved in type III secretion [30], was the only virulence factor which showed significantly increased expression in the Prn-Neg strains. For the Prn-Pos strains hemC, involved in iron uptake, ctpA, involved in the maturation of FHA, and Prn were significantly higher expressed. No differences in the expression of major virulence factors, such as adenylate cyclase toxin and dermonecrotic toxin, were observed between Prn-Pos and Prn-Neg strains (Table S6). Interestingly, metabolic related proteins, such as arsC and aceF (Figure 5(b), Table S6), were found significantly upregulated in Prn-Neg strains, while other metabolic related proteins, such as thrB and BP2338, were significantly higher expressed in the Prn-Pos strains, suggesting differences in metabolism between the *B. pertussis* Prn groups.

## Discussion

*Bordetella pertussis* strains that do not express the vaccine component Prn are increasingly circulating in countries that use aP vaccines. Adaptation of *B. pertussis* to evade vaccine induced immunity is a plausible explanation for the emergence of these Prn-deficient strains. In addition to evading Prn-specific bactericidal antibodies [6] we were interested to investigate whether these Prn-deficient *B. pertussis* strains could also induce a different innate immune activation compared to their Prn-expressing counterpart.

Our findings indicate that moDCs stimulated with Prn-Neg *B. pertussis* strains secreted higher levels of proinflammatory cytokines and showed enriched expression of immune activation associated genes as compared to moDCs stimulated with Prn-Pos strains. This is in agreement with our previous work which showed that Prn KO *B. pertussis* strains induce enhanced proinflammatory cytokine secretion by moDC [9]. However, no significant difference in the secretion of the anti-inflammatory cytokine IL-10 was observed, which was also reported by Stefanelli et al. [9,31]. Interestingly, the increase of proinflammatory cytokine secretion was much stronger when using the Prn-KO strain compared to the naturally circulating Prn-Neg strains. This made us question whether in these naturally circulating strains, proteins other than Prn were differentially expressed. Indeed, the whole cell proteome analysis of the nine *B. pertussis* strains performed in the current work, showed distinct protein expression profiles associated with Prn-Pos and Prn-Neg strains, revealing other proteins which can potentially play a role in the differential activation of moDCs. One of the proteins showing higher expression in the Prn-Neg strains is a pyruvate dehydrogenase (aceF) which has been shown to confer resistance to stress and enhance survival in other bacterial pathogens [32]. Future studies should investigate the function of these differentially expressed proteins and their role in moDC activation as this can give insight into the evolution process of Prn-deficient *B. pertussis* strains.

We also performed a transcriptomic analysis of *B. pertussis*-stimulated moDCs to unravel immunological processes induced by *B. pertussis*. This analysis revealed clear activation of moDCs by all *B. pertussis* strains and significant differences in the gene expression of Prn-Pos and Prn-Neg stimulated moDCs. The majority of fold changes from the differentially expressed genes between the two Prn (Pos and Neg) groups was below the typically used threshold of 1.5. This indicates that after 3 h of moDC stimulation with the *B. pertussis* strains, significant but subtle differences in gene expression were observed. Future studies should explore gene expression of moDC upon exposure to *B. pertussis* at different time points as it has been shown for other innate cells that this can change in time [33]. Nevertheless, despite the modest increased expression of *CSF3*, *IL12B* and *CXCL8* in moDC stimulated with Prn-Neg compared to the Prn-Pos strains, we observed a significantly increased secretion of their gene products, G-CSF, IL-12p70 and IL-8, respectively. This showed that the subtle differences in gene expression had a significant effect on protein translation and secretion of moDCs upon stimulation with these bacterial strains.

One of the most striking observations from the transcriptomic data is the enrichment of IFNα response associated genes in Prn-Pos stimulated moDCs. In contrast, expression of genes associated with TNF signaling via NF-κB and Interleukin (JAK/STAT3) signaling were enriched in Prn-Neg stimulated moDCs, suggesting the induction of distinct inflammatory pathways by these two *B. pertussis* groups. Type I IFN responses can have protective but also detrimental effects during bacterial infections [34,35]. Recent work in IFNAR1-SA mice revealed that increased type I IFN signaling during a *B. pertussis* infection leads to exacerbated lung pathology in adult mice [36]. In contrast, this same study showed significantly lower levels of lethality in infant IFNAR1-SA mice infected with *B. pertussis* as compared to infant wild type mice. This indicates that type I IFN signaling has an age-dependent beneficial or detrimental effect during *B. pertussis* infection. Another study in mice revealed that plasmacytoid DCs in the lungs of *B. pertussis* infected mice produce high levels of IFNα and that this inhibits the rapid increase in Th17 cells [37]. As both of these studies made use of Prn-expressing *B. pertussis* strains, the question remains if infection with Prn-deficient *B. pertussis* would alter lung pathology or early Th17 differentiation and whether these processes also occur in humans.

Another remarkable finding was the enrichment in gene expression associated with oxidative phosphorylation and fatty acid metabolism in the Prn-Pos stimulated moDCs. These set of genes have been reported to be associated with resting DCs [38]. In contrast, Prn-Neg stimulated moDCs showed enriched expression of genes associated with proliferation, inflammatory responses and protein secretion. All these enriched gene sets hint towards enhanced immune activation of human moDCs stimulated by Prn-Neg *B. pertussis* which was corroborated with the cytokine secretion profile of these cells. Although it has been shown that Prn-deficient *B. pertussis* strains showed improved entry into human moDCs and epithelial cells [31,39], we did not find any significant difference in gene expression associated with bacterial entry nor intracellular bacterial sensing. As timing of our transcriptome analysis may influence these findings, future studies should include transcriptomics of these cells at different time points.

Despite the enhanced proinflammatory moDC activation by Prn-deficient strains, they do not appear to induce enhanced disease compared to the Prn-expressing strains [40]. Yet, whether the enhanced immune activation by these strains is beneficial for bacterial survival *in vivo* remains to be determined. In this regard, in Japan there was a significant reduction in the amount of circulating Prn-deficient strains after switching to an aP vaccine that did not contain Prn [41]. This would suggest that expressing Prn is beneficial for *B. pertussis* to establish infection unless the bacterium is circulating in a population vaccinated with a Prn-containing aP vaccine. It has become clear that Prn-deficient *B. pertussis* strains successfully circulate in populations vaccinated with Prn-containing aP vaccines. This highlights the importance of considering pathogen adaptation when designing new pertussis vaccines as the induced genetic changes in this bacterium could alter immune responses to this pathogen and favour their circulation.

Altogether, our data shows that Prn-Neg *B. pertussis* strains induce a distinct immune activation profile of moDCs and that different bacterial protein expression profiles were associated with Prn-Pos and Prn-Neg strains. This suggests that Prn-deficiency in *B. pertussis* is not only relevant to evade antibody-mediated immunity induced by the aP vaccine, but also that these emerging strains can alter hosts cellular immune responses. This adds a novel dimension to the role of pathogen adaptation in the reemergence of bacterial pathogens after vaccination and highlights the complexity of host-pathogen interactions. We show that combining data of naturally circulating *B. pertussis* strains with that of immune responses against these emerging strains is essential to fully track the effect vaccine pressure has on *B. pertussis* adaptation.

## Supplementary Material

Supplementary_materials_Kroes_et_al_for_submission_EMaI_Revised.docx

## Data Availability

All RNAseq data is available in the GEO database, accession number: GSE164643.
