# Supplementary material for: Naturally circulating pertactin-deficient Bordetella pertussis strains induce distinct gene expression and inflammatory signatures in human dendritic cells
Source: Emerg Microbes Infect. 2021 Jul 5;10(1):1358–68. doi: 10.1080/22221751.2021.1943537 (PMC8259873; doi:10.1080/22221751.2021.1943537)
Supplement: Supplementary_materials_Kroes_et_al_for_submission_EMaI_Revised.docx [file TEMI_A_1943537_SM8683.docx]

**Supplementary materials**

# **Tables**

Table S1. ***B. pertussis* strains used in this study.** ID indicates strain identification letter

| **Strain** | **ID** | **Year of isolation** | **MLVA** | **ptxP type** | **Prn type** | **ptxA type** | **fim2** | **fim3** | **Prn mutation** |
| --- | --- | --- | --- | --- | --- | --- | --- | --- | --- |
| **B4436** | A | 2016 | 27 | 3 | 2 | 1 | 1 | 2 | IS481 (Ba) |
| **B4414** | B | 2016 | 27 | 3 | 2 | 1 | 1 | 1 | Inversion C |
| **B4442** | D | 2016 | 27 | 3 | 2 | 1 | 1 | 1 | Inversion C |
| **B4393** | F | 2016 | 27 | 3 | 2 | 1 | 1 | 1 | Intact |
| **B4430** | G | 2016 | 27 | 3 | 2 | 1 | 1 | 1 | Intact |
| **B4431** | H | 2016 | 27 | 3 | 2 | 1 | 1 | 1 | Intact |
| **B1707** | J | 1998 |  | 3 | 2 | 1 | 1 | 1 | Intact |
| **B1805** | K | 1998 |  | 3 | 2 | 1 | 1 | 1 | Intact |
| **B1960** | L | 1998 | 27 | 3 | 2 | 1 | 1 | 1 | Intact |

Table S2. **List of DEGs in the Unstim-Ctrl vs Prn-Pos comparison.**

Table S3. **List of DEGs in the Unstim-Ctrl vs Prn-Neg comparison.**

Table S4. **List of DEGs in the Prn-Pos vs Prn-Neg comparison.**

Table S5. **Complete list of genes in the BTM analysis (related to Fig 4).**

Table S6. **List of DEPs in the Prn-Pos vs Prn-Neg *B. pertussis*** **strain comparison.**

Table S7. **Statistical analysis of Prn-Pos (2016 + 1998) vs Prn-Neg *B. pertussis* induced cytokine secretion by moDCs.** padj; adjusted p-value

| **Cytokine** | **padj** | **Effect size (Type)** | **Effect size interpretation** |
| --- | --- | --- | --- |
| **IL-12p70** | 1.23E-06 | 1.77 (Hedge’s g) | Very large |
| **TNF** | 4.12E-04 | 0.61 (Cliff’s delta) | Large |
| **G-CSF** | 7.59E-03 | 1.21 (Hedge’s g) | Very large |
| **IL-8** | 3.75E-04 | 1.31 (Hedge’s g) | Very large |
| **IL-6** | 4.69E-01 | 0.45 (Cliff’s delta) | Large |
| **IL-10** | 1.00E+00 | 0.34 (Cliff’s delta) | Medium |

Table S8. **Secreted** **cytokine concentrations of Prn-Pos and Prn-Neg *B. pertussis* stimulated moDCs.** SD; standard deviation.

| **Cytokine** | **Prn-Neg Mean +/-SD (pg/ml)** | **Prn-Pos (2016 + 1998)  Mean +/-SD (pg/ml)** |
| --- | --- | --- |
| **IL-12p70** | 23 +/-16 | 14 +/-10 |
| **TNF** | 6540 +/-4411 | 5093 +/-3052 |
| **G-CSF** | 64 +/-40 | 45 +/-22 |
| **IL-8** | 5465 +/-1495 | 4709 +/-1590 |
| **IL-6** | 418 +/-399 | 344 +/-312 |
| **IL-10** | 277 +/-281 | 190 +/-154 |

# **Materials and Methods**

## ***Bacterial strain selection***

Nine *B. pertussis* strains, isolated from Dutch pertussis patients, were selected for use in this study (Table S1). Six of the selected strains expressed intact Prn while three of the strains were Prn-deficient. To minimize variation between the *B. pertussis* strains other than Prn expression, the strains were all selected from the *PtxP3* lineage which is the predominantly circulating lineage in the Netherlands [1] and the strains were matched, as well as possible, on *Prn*, *ptxA*, *fim2* and *fim3* allele type, which were previously determined as described by Mooi et al. 2000 [2]. Furthermore, all Prn-Neg and three of the Prn-Pos strains were isolated in 2016 while the remaining three Prn-Pos strains were strains isolated in 1998. All strains showed comparable growth rate.

## ***Determination of antigen production by B. pertussis using whole-cell ELISA***

Bacterial strains were inoculated on to Bordet Gengou agar plates, supplemented with glycerol and 15% defibrinated sheep blood (BD Biosciences), and grown at 35⁰C and 5% CO_2_ for 4 days. Subsequently, bacteria were suspended in THIJS medium [3] at OD_600_ 0.15 and cultured for 14 h at 35°C rotating at 180rpm. An immulon 2HB flat-bottom 96-well plate (Thermo Fisher Scientific) was coated with live *B. pertussis* at OD_600_ 0.1, optimal concentration determined by coating serial dilutions. The various antigens were targeted with HRP-conjugated antibodies. The presence of *B. pertussis* antigens from the various strains is indicated as the OD_450_ measurement of HRP-converted TMB substrate after stopping the reaction by addition of H_2_SO_4_.

## ***RNA isolation and RNA sequencing***

For RNA isolation the column-based miRNeasy RNA isolation kit (Qiagen) was used according to the manufacturer’s instructions. To determine RNA quality and integrity we used Lab-on-Chip analysis on an Agilent 2100 Bioanalyzer (Agilent Technologies). RNA samples with an RNA integrity number above 8 were used. TruSeq Stranded mRNA Library Prep Kit (Illumina) was used to prepare libraries for the Illumina platform following manufacturer’s instructions, using 1 µg of total RNA as input. The sequencing was performed with the Illumina NextSeq 500/550 High Output Kit v2.5 (single-end, 75 Cycles). Samples were randomized and processed in 4 sequencing runs. Basecalling and demultiplexing was performed using bcl2fastq2 Conversion Software v2.20, and demultiplexed FASTQ files were generated based on sample-specific barcodes (>15 million reads/sample).

## ***Statistical analysis***

Supernatant cytokine output was normalized by dividing each measurement by the mean value of all measurements. The best variables that explained the data were selected with the aid of *leaps* R package. For all cytokines, a linear model was fitted, and its assumptions were checked, namely normality of the residuals (*shapiro.test()* from *stats* R package), homoscedasticity (*bptest()* from *lmtest* R package), independence of errors (*durbinWatsonTest()* from *car* R package) and the absence of outliers (*outlierTest()* from *car* R package). If any of the assumptions were not held for any cytokine (*p*-value <0.05 in any of the tests), a power transformation was done with the *lambda* coefficient (*powerTransform()* from *car* R package), reassessing the assumptions in the same way. Just for those cytokines passing the assumption tests, direct or power transformed, a linear model was applied to detect differences between Prn groups, concretely Prn-Pos vs Prn-Neg, and these groups against Unstim-Ctrl. If assumptions didn’t hold, an Exact Wilcoxon-Mann-Whitney test, which is a permutation based test, was applied. All the *p*-values were corrected for multiple testing by the Bonferroni-Hochberg method (padj).

Effect sizes (ES) were calculated, providing *Hedges’g* [4] for the parametric analysis and Cliff’s delta [5] for the non-parametric analysis (function *mes2()* from *compute.es* R package). The descriptors for Hedge’s g are the following: small, if <0.2; medium if <0.5; large, if <0.8; very large, if <1.2; and huge, if <2 [6]. For Cliff’s delta, the descriptors are: small, <0.28; medium, <0.43; large, >= 0.43 [7]. All comparisons with a padj <0.05 and ES medium or higher are considered as biologically relevant in the present work.

# **References Supplementary File**

1. Mooi FR, van Loo IH, van Gent M, et al. *Bordetella pertussis* strains with increased toxin production associated with pertussis resurgence. Emerg Infect Dis. 2009 Aug;15(8):1206-13.

2. Mooi FR, Hallander H, Wirsing von König CH, et al. Epidemiological Typing of Bordetella pertussis Isolates: Recommendations for a Standard Methodology. European Journal of Clinical Microbiology and Infectious Diseases. 2000 2000/04/01;19(3):174-181.

3. Thalen M, IJssel J, Jiskoot W, et al. Rational medium design for Bordetella pertussis: basic metabolism. J Biotech. 1999;75:13.

4. Hedges LV. Distribution Theory for Glass's Estimator of Effect size and Related Estimators. Journal of Educational Statistics. 1981;6(2):107-128.

5. Cliff N. Dominance statistics: Ordinal analyses to answer ordinal questions. Psychological Bulletin. 1993;114(3):494-509.

6. Sawilowsky SS. New Effect Size Rules of Thumb. Journal of Modern Applied Statistical Methods. 2009;8(2):597 - 599.

7. Vargha A, Delaney HD. A Critique and Improvement of the CL Common Language Effect Size Statistics of McGraw and Wong. Journal of Educational and Behavioral Statistics. 2000;25(2):101-132.
